# Oral mycotic infection caused by a rare *Verticillium* species – a case report

**DOI:** 10.1186/s12903-023-03128-2

**Published:** 2023-07-02

**Authors:** Saqib Habib, Nighat Naved, Muhammad Sohail Awan

**Affiliations:** 1 Operative Dentistry & Endodontics, Aga Khan University Hospital, Karachi, Pakistan; 2 Department of Surgery, Aga Khan University Hospital, Karachi, Pakistan

**Keywords:** Deep mycotic infection, Verticillium species, Rare fungus, Immunocompromised individuals, Histopathological evaluation, Oral rehabilitation

## Abstract

**Background:**

Deep-seated fungal infections of the oral cavity present a diagnostic challenge as the clinical presentation is usually aggressive leading to misdiagnosis of malignancy. Nevertheless, the species of fungi responsible for such diseases in immunocompromised individuals are varied thus, further complicating the diagnosis.

**Case presentation:**

Presented below is a case regarding the diagnosis and management of deep mycotic infection of the oral cavity caused by a fungus that very rarely causes disease in humans, the *Verticillium* species.

**Conclusions:**

The case highlights the fact that rare pathogens should also be considered in the differential diagnosis, especially in patients with debilitating conditions like uncontrolled diabetes. Likewise, histopathological evaluation and microbiological investigations are of paramount importance and remain the gold standard to reach a definitive diagnosis.

## Introduction

Immunocompromised patients, as well as those with debilitating conditions like uncontrolled diabetes, neoplastic disease, and organ transplants, are at increased risk of opportunistic fungal infections [1]. The oral cavity, on the other hand, in the presence of any metabolic disorder like diabetes mellitus may present with xerostomia, thereby allowing these organisms to thrive [2]. Thus, any fungus present in the oral environment can be potentially pathogenic in such patients.

Oral mycosis or fungal infections can be further categorized into superficial and deep. The latter rarely involves oral cavity; however, it may occur in immunocompromised individuals [3]. Deep-seated mycotic infections present a diagnostic challenge as the clinical presentation is varied and usually aggressive, involving deeper penetration of pathogens resulting in ulceration or perforation of the bony areas, hence, mimicking malignancy [4].

Very few fungal species like Histoplasma, Mucor, Blastomyces, and Chromoblastomycoses causing widespread diseases in humans have been reported in literature to cause deep-seated infections of the oral cavity along with systemic involvement [3]. Having said that, presented below is a case of deep mycotic infection of the oral cavity caused by a fungus that very rarely causes disease in humans, the *Verticillium* species.

## Case presentation

A 48-year-old female known case of diabetes mellitus, presented to the emergency department of Aga Khan University Hospital, Karachi in January 2019, with the complaint of swelling on right side of face along with bleeding and maggots coming from nose for the past few days.

Upon inquiring about detailed history, the patient was in a usual state of health, when one morning immediately after getting up from sleep she experienced an episode of nasal bleed. Accompanied by bleeding, was low-grade fever with rigors, facial swelling, headache, pain as well as difficulty in mouth opening. For headache and facial pain, she went to a general physician where over-the-counter painkillers were administered which provided temporary relief. She had a similar episode of facial swelling two years back, which was diagnosed as an endodontic abscess that resolved when the offending teeth were extracted. Furthermore, she reported multiple extractions of teeth in the past years, however, the patient did not recall the reason and the exact timings for extraction.

**Fig. 1:**
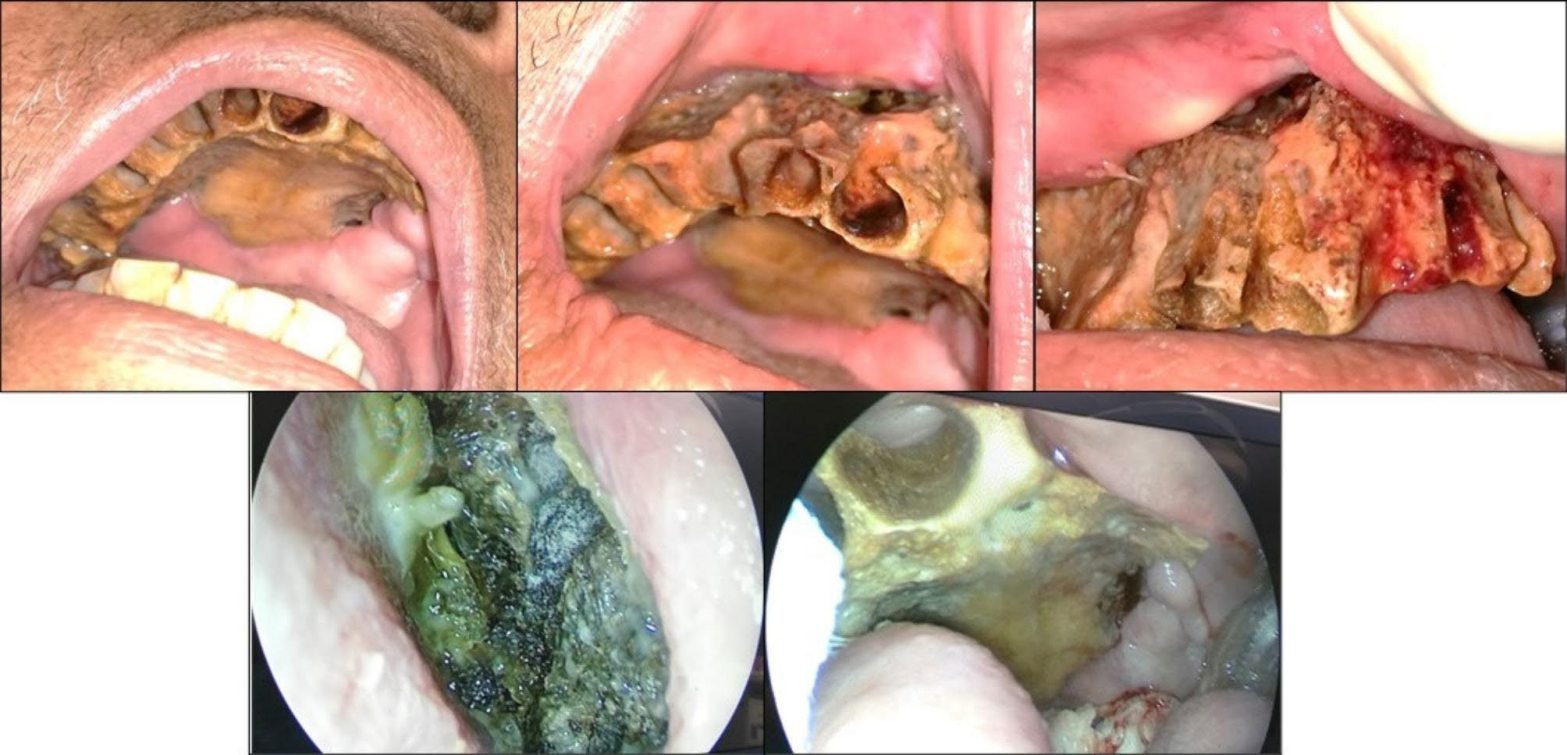
Pre-operative pictures and an endoscopic view showing unhealed extraction sockets, necrotic maxillary alveolus, and right side of the palate

Extraoral examination revealed facial asymmetry secondary to swelling on the right side of the face which was tender on palpation. On intraoral examination, the patient had foul-smelling breath with completely exposed and necrotic maxillary alveolus and the right side of the palate. Moreover, multiple non-healed alveolar sockets were observed in the maxilla. Nasal endoscopy revealed crusted and dried mucosa with an exposed nasal septum and the right lateral wall of the nose (Fig. 1).

To investigate the extent of necrosis, a Computed Tomography (CT) scan of the head and neck was ordered, which showed mucosal thickening in bilateral ethmoid, sphenoid, maxillary and frontal sinuses with air specks, some enhancement, erosion, and destruction of the right frontal bone, nasal septum, right lamina papyracea, and hard palate. No established infarct, intracranial hemorrhage, or mass effect was noted otherwise. Few sub-centimeters enhancing level I, II, and V cervical lymph nodes were noted. The largest level II lymph node measured 7.8 mm on the left side and approximately 6 mm on the right side.

Based on the patient’s initial findings, a differential diagnosis of maxillary osteomyelitis, mucormycosis, or fungal sinusitis was made.

For basic laboratory blood workup, a blood sample was sent for laboratory investigations which revealed an elevated leucocyte count of 17.4 × 10^9^/L, hemoglobin of 9.9 with reduced hematocrit 32%, glycated hemoglobin (HBA1c) of 9.5, significantly elevated erythrocyte sedimentation rate (ESR) 99 mm/hr, C- reactive protein (CRP) 19.82 mg/dl, and blood urea nitrogen (BUN) 35 mg/dl with creatinine of 1.8 mg/dl. The right nasal swab was sent for culture where moderate colonies of cloxacillin resistant-Staphylococcus Aureus were identified, whereas it was negative for any fungal growth.

Thus, comprehending all findings, the patient was started on intravenous (IV) vancomycin since the identified organism was resistant to all beta-lactam antibiotics. Serum trough concentrations were obtained prior to the fourth and fifth doses to guide the level of therapeutic concentration. Likewise, insulin therapy was administered to address uncontrolled diabetes. Moreover, an otolaryngologist and maxillofacial surgeon were taken on board for the debridement of necrotic maxilla and nasal cavities.

Under the care of otolaryngology, a biopsy specimen was obtained. The smear revealed few septate hyphae, and the culture came out positive for Aspergillus flavus however, this time no bacteria could be identified. The patient was started on IV Amphotericin B 50 mg once daily for 10 days and the patient’s family was counseled regarding the need for external debridement of the necrotic maxilla and Functional Endoscopic Sinus Surgery (FESS) under general anesthesia.

**Fig. 2:**
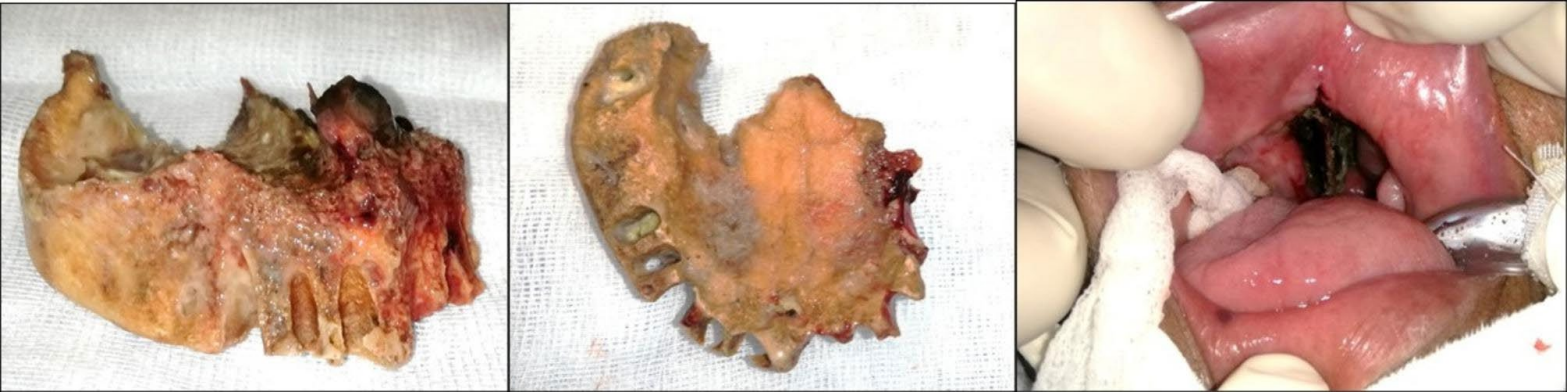
Dorsal and ventral view of the excised palate and post-operative intraoral view

After informed consent was obtained, external debridement of the maxilla, hard palate, and partial ethmoidectomy along with FESS was performed under general anesthesia (Fig. 2). The extent of debridement was made to the point till healthy soft and hard tissue was observed clinically. The surgical site was copiously irrigated with normal saline, and the specimen was sent for histopathological and microbiological evaluation.

**Fig. 3:**
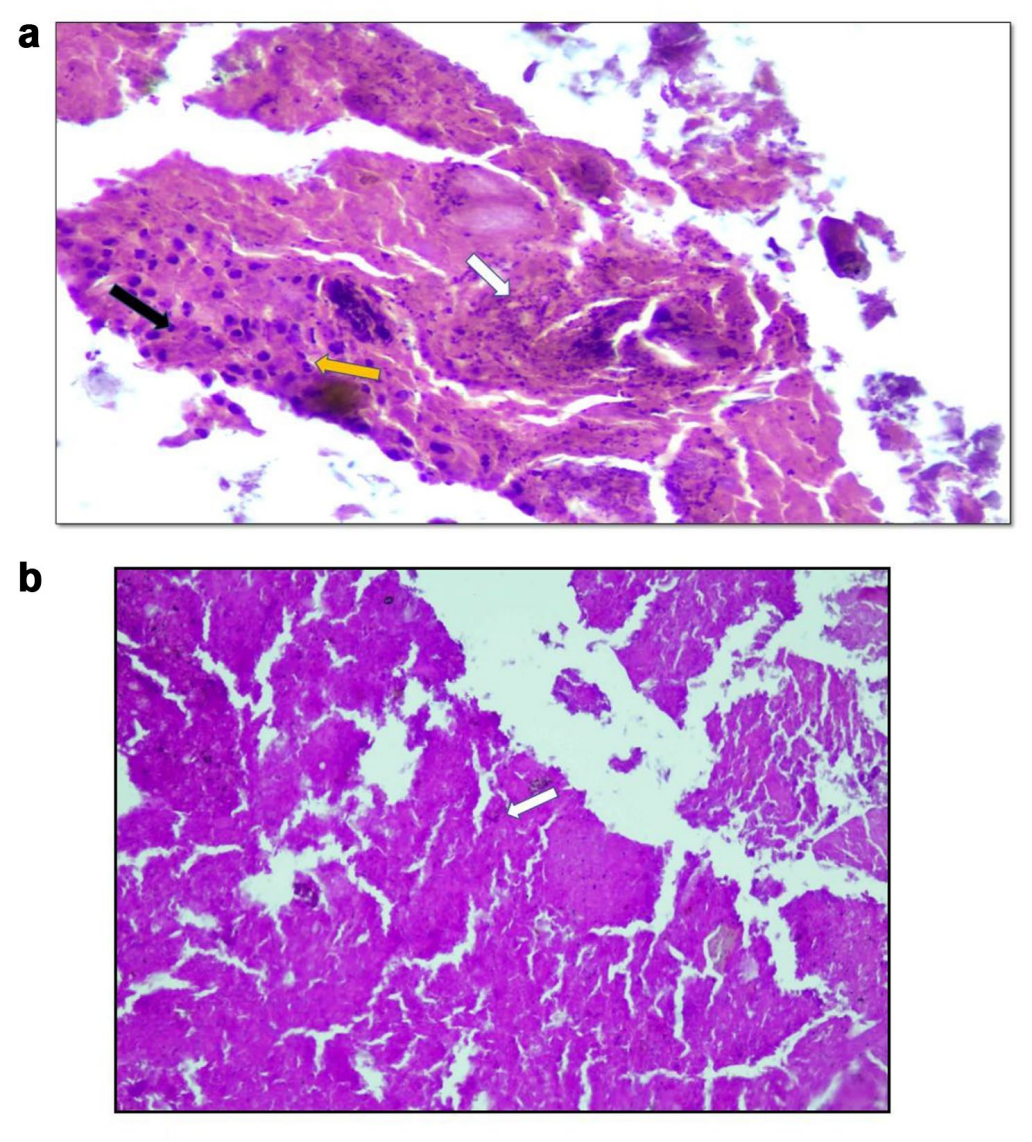
**(a)** Microscopic examination of the tissue stained with hematoxylin and eosin. The black arrow shows lymphocytes, the yellow arrow shows neutrophils, and the white arrow shows fungal spores. **(b)** Microscopic examination tissue stained with Periodic Acid-Schiff stain with Diastase (PAS-D). The white arrow shows the septate fungal hyphae

Histopathologic examination of the specimen with hematoxylin and eosin revealed multiple fragments of bone along with acute and chronic inflammatory cells. The intertrabecular areas exhibited foci of necrosis and hyalinization whereas scattered hyphae of fungal organisms with focal septation were seen as shown in (Fig. 3a). Special stain i.e., Periodic Acid Schiff with Diastase (PAS-D) was used for the identification of fungal spores, which showed few septate hyphae as shown in (Fig. 3b). Furthermore, the specimen was sent for microbiological culture to correlate with the histopathological findings. For fungal growth, a culture medium i.e., potato dextrose agar was used, which showed negative growth for up to two weeks. After four weeks, the final culture was positive for the growth of a rare fungus – *Verticillium* species.

Post-operatively, the patient was discharged in a vitally stable condition and advised to perform nasal douching with normal saline at least five times daily. She was asked to continue taking prescribed antibiotics and antifungals (IV Piperacillin-Tazobactam 2.25 gm thrice daily, Tab Voriconazole 400 mg twice daily for seven days) and was referred to a maxillofacial prosthodontist for rehabilitation of maxillary defect.

At two weeks follow-up period, a healthy intra-oral and nasal mucosa was observed with no post-operative complications. The patient was advised to get a CT scan for assessment of postoperative healing, but she did not provide consent for the imaging. To monitor the patient regularly, six monthly follow-up visits were advised, during which she was disease-free. For the rehabilitation of the maxillary defect with an obturator, she did not consult a maxillofacial prosthodontist due to financial constraints, hence no further intervention was provided.

## Discussion

*Verticillium*, a filamentous fungus, is a major plant pathogen that very rarely causes disease in humans. Only a few case reports have implicated the role of *Verticillium* species in causing fungemia, keratitis, skin infection, endophthalmitis, peritonitis as well as hepatosplenic abscess in humans [5–10]. However, to the best of our knowledge, this is the first report presenting a case of deep mycotic infection of the oral cavity caused by *Verticillium* species.

Patients with uncontrolled diabetes mellitus are at high risk for acquiring fungal infections owing to poor glycemic control. This eventually leads to additional complications such as reduced salivary flow and pH as well as increased salivary glucose, resulting in the opportunistic growth of fungi and bacteria [2]. Thus, an early and accurate diagnosis is the cornerstone for effective management in such clinical scenarios.

The patient when presented to the hospital, had acute signs of infection along with facial pain and swelling, based on these observations a differential diagnosis of maxillary osteomyelitis or oral mycotic infection was made. The patient was initially started on IV vancomycin as the preliminary culture results yielded cloxacillin-resistant bacterial colonies. However, the patient was shifted to Amphotericin B as soon as the culture came out positive for fungal species, Aspergillus flavus, and a rare one *Verticillium*.

Amphotericin B is a broad-spectrum antifungal agent, but is associated with nephrotoxicity [11]. Having said that, our patient had acute kidney injury (AKI) secondary to uncontrolled diabetes therefore, the liposomal formulation of Amphotericin B was administered which is less nephrotoxic (14.5%) compared to the conventional one (32.5%) as reported by a meta-analysis [12]. Nevertheless, in patients receiving drugs with potential renal effects, high-quality supportive care should be provided to maintain the tubular function of kidneys. Thus, the acid base balance, fluids and electrolytes of the patient were strictly monitored during the entire course of treatment.

The case highlights the fact that rare pathogens should also be considered in the differential diagnosis, especially in patients with debilitating conditions like uncontrolled diabetes mellitus. Likewise, histopathological evaluation and microbiological investigations are of paramount importance and remain the gold standard in order to reach a definitive diagnosis [1]. Therefore, the specimen should be sent for culture as soon as possible so that an appropriate course of action is followed at the right time.

It is evident in the literature that all cases associated with *Verticillium* species have been reported in immunocompromised patients therefore, it is important that proper head and neck screening of such patients should be routinely performed at frequent intervals [6–8]. Likewise, patients should be counseled regarding the consequences of certain metabolic disorders like uncontrolled diabetes and should be encouraged to maintain a healthy and balanced life to minimize the occurrence of such debilitating conditions.

## Conclusion

Deep-seated fungal infections of the oral cavity present a diagnostic challenge as the clinical presentation is usually aggressive leading to misdiagnosis of malignancy. Nevertheless, the species of fungi responsible for such diseases in immunocompromised individuals are varied thus, further complicating the diagnosis. Therefore, histopathological, and microbiological investigations should be done in all cases as soon as possible for accurate diagnosis as well as management which would certainly improve the patient’s quality of life.

## Data Availability

All data generated or analyzed during the course of treatment are included in this published article.
